# Supplementary material for: Co-occurrence of non-communicable disease risk factors and its determinants among school-going adolescents of Kathmandu Metropolitan City
Source: PLoS One. 2022 Aug 10;17(8):e0272266. doi: 10.1371/journal.pone.0272266 (PMC9365171; doi:10.1371/journal.pone.0272266)
Supplement: S1 Table — (DOCX) [file pone.0272266.s001.docx]

**S1 Table.** **Univariate analysis to determine factors associated with co-occurrence of NCDs risk factors**

| **Variables** | **No risk factors, n(%)** | **One risk factors, n(%)** | **Co-occurrence of risk factors, n(%)** | **P-value** |
| --- | --- | --- | --- | --- |
| **Sex** |  |  |  |  |
| Male | 96 (15.9) | 262(43.4) | 246(40.7) | 0.192 |
| Female | 62(12.3) | 237(47.0) | 205(40.7) |  |
| **Age category** |  |  |  |  |
| 13-16 | 100(15.3) | 306(46.9) | 247(37.8) | 0.059 |
| 17-19 | 58(12.7) | 193(42.4) | 204(44.8) |  |
| **Grade** |  |  |  |  |
| Secondary | 88(13.9) | 296(46.6) | 251(59.5) | 0.473 |
| Higher Secondary | 70(14.8) | 203(42.9) | 200(42.3) |  |
| **Ethnicity** |  |  |  |  |
| Brahmin/Chhetris | 84(16.4) | 256(49.9) | 173(33.7) | **<0.001** |
| Other | 74(12.4) | 243(40.8) | 278(46.7) |  |
| **Religion** |  |  |  |  |
| Hindu | 119(13.5) | 410(46.4) | 354(40.1) | 0.125 |
| Other | 39(17.3) | 89(39.6) | 97(43.1) |  |
| **Currently living with** |  |  |  |  |
| Both parents | 103(14.8) | 317(45.5) | 277(39.7) | 0.650 |
| Other than parents | 55(13.4) | 182(44.3) | 174(42.3) |  |
| **Close friends** |  |  |  |  |
| **Fewer than 3** | 64(13.0) | 214(43.6) | 213(43.4) | 0.535 |
| **At least 3** | 94(15.2) | 285(46.2) | 238(38.6) |  |
| **Fathers’ education** |  |  |  |  |
| Secondary and below | 102(13.7) | 339(45.4) | 306(42.0) | 0.499 |
| Higher secondary and above | 56(15.5) | 160(44.3) | 145(40.2) |  |
| **Mothers’ education** |  |  |  |  |
| Secondary and below | 109(12.7) | 377(43.8) | 374(43.5) | **<0.001** |
| Higher secondary and above | 49(19.8) | 122(49.2) | 77(31.0) |  |
