# Supplementary material for: Co-occurrence of non-communicable disease risk factors and its determinants among school-going adolescents of Kathmandu Metropolitan City
Source: PLoS One. 2022 Aug 10;17(8):e0272266. doi: 10.1371/journal.pone.0272266 (PMC9365171; doi:10.1371/journal.pone.0272266)
Supplement: Attachment — Submitted filename: Response to Reviewers (2).docx [file pone.0272266.s003.docx]

Overall comment:

1) We note you have included a table to which you do not refer in the text of your manuscript. Please ensure that you refer to Tables 2 and 4 in your text; if accepted, production will need this reference to link the reader to the Table.

**Tables are correctly indicated in the text**

2) Please include a copy of Table 12 which you refer to in your text on page 16.

**Table 12 was typographical error which is corrected.**

**Reviewer 1**

1) The author points out in the study design that the age range of the subjected students is 13-19 years old, but 10-14 years old appears in Table 2, and 17-20 years old appears in line 234. The age definition and segmentation standards of the research objects are too confusing.

**The difference in age category label between methods, Table 2 and line 234 is because of typographical error which is corrected after checking the dataset which is available in the open data registry.**

2) According to Table 1, Janajati has the highest share of Ethnicity then why is Brahmin the definition of Ethnicity in Table 4?

**The Janajati, Dalit and Madhesi groups considered as ethnic minorities and they share nearly similar characteristics (like drinking, non-vegetarian diet etc) but does not match with the Brahmin Chhetri ethnic groups. Hence Brahmin/Chhetri group is kept as separate group.**

3) In line 103, it is clear that since belonging to a public or private school will affect the content of this study, the proportion of students should be 1:1. This may bring great error to the research results of this paper.

**The descriptive analysis is added stratified by type of school (public/private). In multivariate regression, the type of school is also adjusted.**

4) In line 224, after the description of the data, the conclusion "Two-fifths of the school-going adolescents had co-occurrence of any two or more non-communicable disease risk factors" should be clearly stated so that the reader can understand the conclusions in the abstract.

**It is clarified in the abstract and in result section.**

**Reviewer 2**

1) Abstract: Please make sure that the conclusion completely follows the idea behind the study and reflects the main findings.

**Conclusion part in abstract is edited so that it follows the idea behind the study and main findings.**

2) Introduction: The main focus of the introduction must be on the NCDs among adolescents. The majority of the introduction is out of the funnel of the storyline of the introduction. I highly recommend to revise the section.

**Relevant Content are added to the introduction part**

3) Discussion: It is necessary to mention the authors' understanding of the article findings well. Please review the discussion, check its storyline, and improve its coherency. It is not easy to follow in its current form.

**Discussion has been revised as per suggestion.**

**Reviewer 3**

1. This study is cross-sectional, which only reveals the association. Also, the study has not any timing-based followed-up. So, the observed results only reveal the association, which is not sufficient. Based on current study, we could not conclude the accurate non-communicable disease risk factors.

**I agree with you that cross-sectional study is not appropriate to determine risk factors. In this study we have determined factors associated with co-occurrences of different established NDCs risk factors, not the factors associated with NCDs. We have used STEPS survey to find the whether the school going adolescents have four different NCDs risk factors or not.**

2. The definition of the terms that are assessed in the method section is determined without any references, which provides additional biases for the way that authors have defined them.

**References are now added to the definition of terms in method section**

3. Providing a figure regarding the results of study will help to convey the content of the study more sufficiently.

**Figures are added to the manuscript**

**Reviewer 4**

1. Limitations of the study: The authors did well to mention the limitations of the study but they fell short of suggesting how these limitations should be addressed by future studies going forward

**We have revised the limitations as per your suggestion.**

2. No inclusion/exclusion criteria were stated

**Inclusion and exclusion criteria are added in the participant recruitment section of manuscript**

3. References: The manuscript employed the use of Harvard style referencing but requires editing to correct some errors noticed e.g., listing of references: Shouldn’t this be in alphabetical order? Shouldn’t the journal name be italics? Shouldn’t the list of authors that are more than 5 be reflected as et al? I suggest the authors should revise Harvard referencing style and make necessary corrections.

**We used Vancouver referencing style specific to PLOS One as referencing guideline.**

**Reviewer 5**

1) The background section needs a clear explanation for the study

**Background is modified for clear explanation**

2) The methodology section is lacking and requires more information about the analysis process (e.g., how were the variables selected for the multivariate model)

**It is added in the manuscript. In the model we added socio-demographic variables- age, gender, ethnicity, religion, education, type of school, and parental education (mother’s education, and father’s education) with p-value less than 0.50 in univariate analysis (S 1 Table of supplementary file).**

3) The results sections need better organization guided by a clear framework. Due to the limited scope of the findings presented in the paper, it does not add more value to the existing evidence.

**The results are now modified.**
